# Thematic coverage and readability of online patient information on cochlear implant care

**DOI:** 10.1007/s00405-024-08694-x

**Published:** 2024-05-06

**Authors:** Anna Levi, Martin Leinung, Silke Helbig, Daniela Guderian, Christian Issing, Tobias Weissgerber, Maria Hartmann, Timo Stöver, Andreas G. Loth

**Affiliations:** Department of Otorhinolaryngology, Goethe University Frankfurt, University Hospital, Theodor Stern Kai 7, 60590 Frankfurt am Main, Germany

**Keywords:** Cochlear implantation, Quality, Internet search, Patient information, Readability, Thematic coverage

## Abstract

**Introduction:**

The treatment of patients with a cochlear implant (CI) is usually an elective, complex and interdisciplinary process. As an important source of information, patients often access the internet prior to treatment. The quality of internet-based information regarding thematic coverage has not yet been analysed in detail. Therefore, the aim of this study was to analyse the information on CI care available on the internet regarding its thematic coverage and readability.

**Material methods:**

Eight search phrases related to CI care were defined as part of the study. A checklist for completeness of thematic coverage was then created for each search phrase. The current German CI clinical practice guideline and the white paper on CI care in Germany were used as a basis. As a further parameter, readability was assessed using Flesch Reading Ease Scores. The search phrases were used for an internet search with Google. The first ten results were then analysed with regard to thematic coverage, readability and the provider of the website.

**Results:**

A total of 80 websites were identified, which were set up by 54 different providers (16 providers were found in multiple entries) from eight different provider groups. The average completeness of thematic coverage was 41.6 ± 28.2%. Readability according to the Flesch Reading Ease Score was categorised as "hard to read" on average (34.7 ± 14.2 points, range: 0–72). There was a negative statistically significant correlation between the thematic coverage of content and readability (Spearman's rank correlation: r = − 0.413, p = 0.00014).

**Summary:**

The completeness of thematic coverage of information on CI care available on the internet was highly heterogeneous and had a significant negative correlation with the readability. This result should be taken into account by both the providers of internet information and by patients when using internet-based information on CI care and help to further improve the quality of web-based information.

**Supplementary Information:**

The online version contains supplementary material available at 10.1007/s00405-024-08694-x.

## Introduction

Cochlear implants (CIs) are neuroprotheses designed to replace the inner ear function in deaf and severely hearing-impaired patients by electrically stimulating the first neuron of the auditory pathway. Currently, more than 700,000 patients worldwide are treated with a CI [1]. Rehabilitation with a CI is a lifelong process divided into different phases [2]. In the preoperative phase, detailed medical and audiological counseling of patients usually provides the basis for a well-informed decision.

In addition to personalized information gathered at a face-to-face consultation, collection of information from the internet is becoming increasingly important for patients. While the internet search engine Google (Google LLC, Mountain View, USA) reported 1.2 trillion search queries per year in 2012, it was estimated that this figure will rise to more than two trillion in 2021 [3]. In Europe, 42 to 96.4% of all patients have access to the internet [4, 5]. The ever-increasing availability and utilization of web-based information bears considerable opportunities as well as risks [6, 7]**.**

An essential requirement for patients, in order to make meaningful use of internet-based information, is the quality of the information provided. However, this quality consists of several dimensions, e.g. including complete thematic coverage and accuracy of the information as well as readability of the website itself [8–11]. An additional aspect is the traceability of the source that provided the information (creator or provider of the content) [12].

In the medical field, clinical practice guidelines (CPGs) or recommendations for action ("white paper") issued by the leading medical society served as the basis for the assessment of thematic coverage. In Germany, these are the cochlear implant CPG [13] and the white paper on Cochlear Implant Care [2]. Both documents were created under the guidance of the German Society of Otorhinolaryngology, Head and Neck Surgery e.V. (DGHNO-KHC). In terms of medical content, therefore, there is a uniform professional standard in Germany that should represent the basis for patient information.

Among others, an internationally accepted score for evaluating the readability and comprehensibility of texts is the "Flesch Reading Ease Score". This score is a non-dimensional ratio, calculated from the number of syllables, words, and sentences in a text. Higher numerical values indicate easier readability [14].

The aim of this study was to evaluate the quality of websites on key aspects of the CI care process. The quality criteria used were completeness of thematic coverage, readability, and the identification of the provider of the website. Furthermore, we intended to analyze whether there was a statistical correlation between the readability and the thematic coverage of a website.

## Material and methods

**Fig. 1:**
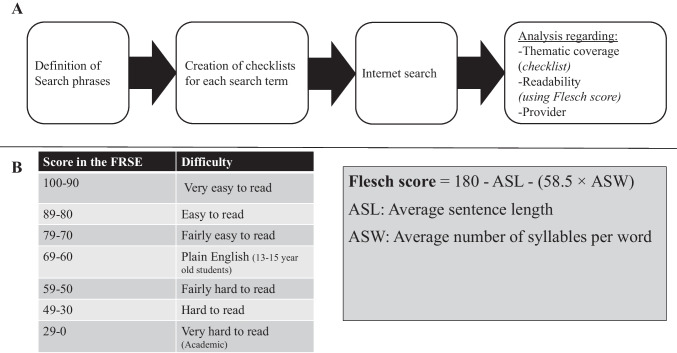
**a** Structure of the present study **b** left: Classification of texts based on the Flesch Reading Ease Score, right: Calculation of the Flesch Reading Ease Score

In this study, no patient-related data were collected or processed; therefore, no approval from an ethics committee was required. The study was conducted in four steps (Fig. 1a). First, the search phrases to be used in the subsequent internet searches were defined. This was followed by the preparation of checklists for each search phrase. The aim of these checklists was to evaluate the completeness of the thematic coverage provided by each website based on the CI CPG and the white book. The third step was to search for the previously defined search phrases using a popular search engine. Finally, the first ten search results of each phrase were analyzed with the check lists and a readability score. Furthermore, the provider of the respective website was documented.

### Definition of search terms

Eight search phrases closely related to the CI care process were defined to conduct the study. These phrases represent relevant aspects based on the currently valid version of the AWMF guideline "Cochlear implant care (AWMF register number 017-071)" and the white paper "Cochlear implant (CI) care" of the DGHNO-KHC [2]. The final search phrases are listed in Table 1.

**Table 1:** List of search phrases used with reference to cochlear implant treatment

Number	Search phrase	Number of possible points of the checklist items
1	Who is suitable for treatment with a cochlear implant	8
2	Counseling process prior cochlear implantation	14
3	Treatment duration of a cochlear implantation	7
4	Contraindications for a cochlear implant treatment	7
5	Surgical risks of cochlear implantation	19
6	Aftercare in cochlear implantation	7
7	Cochlear implantation in children	5
8	Cochlear implants in everyday use	13

### Creating the checklists for completeness of thematic coverage

The thematic coverage of the identified websites was assessed using yes/no style checklists. The maximum number of points for each checklist was achieved if all the items listed for this search phrase (Table 1) in the white paper or CPG were present on the website. The checklist for the search phrase “Risks of CI implantation/surgery” is shown in Table 2 as an example. For this search phrase, 19 different possible surgical risks were stated in the guideline or white paper. Therefore, the checklist had a maximum of 19 points. If an examined webpage were to present all 19 risks, it received 19 points. All checklists are available in the online appendix of this manuscript (Additional file 1).

**Table 2:** Checklist for the search phrase "risks of CI implantation/surgery", which is based on the AWMF guideline "Cochlear implant care (AWMF register number 017-071)" and the white paper "Cochlear implant (CI) care" of the DGHNO-KHC

Number	Checklist item for the search phrase. "Surgical risk of cochlear implantation"	Fulfilled yes/no
1	Infections of the middle ear	
2	Wound healing disorder	
3	Dizziness	
4	Balance disorders	
5	(Permanent) facial nerve paralysis,	
6	Taste disorder	
7	Tinnitus	
8	Loss of any residual hearing	
9	Technical implant failure	
10	Medical complications due to the implant	
11	Need to replace the implant	
12	Deterioration of electrical stimulation, e.g. due to progressive ossification of the cochlea after meningitis	
13	Electrode migration	
14	Unintended stimulation of other cranial nerves (e.g. facial nerve, vestibular nerve)	
15	Incompatibility with implant materials	
16	Hemorrhage; dura and brain injury, cerebrospinal fluid fistula	
17	Failure or absence of the expected hearing success	
18	Neuralgia, scar pain	
19	Anesthesiological risks	

### Internet search

The defined terms (Table 1) were used with the internet engine "Google" [15] between September and December 2021. Various actions were taken to minimize the influence of previous searches on the search results. These measures included searching from different PCs, using the private search mode, and deleting the browser history and cookies after each search. The first ten websites identified for each search were used for further analysis. No pre-filtering was carried out, e.g. for language.

### Analyzing the websites

The identified websites were further analyzed concerning their thematic coverage, readability, and provider of the information. To determine thematic coverage, the websites were assessed using the previously created checklists. A percentage value (0–100%) was calculated in relation to the completeness of the score achieved on the respective checklist.

The readability of the text on each website was determined using the Flesch Reading Ease Score (FES). This score is a standardized, numerical measure of reading comprehensibility, calculated using the average number of words per sentence and the average number of syllables per word [14]. Higher numerical values are equivalent to better readability of a text (Fig. 1b left). The formula used to calculate the German Flesch Reading Ease Score [14] is presented in Fig. 1b.

Subsequently, information available in the imprint regarding the author or provider of the website was collected to identify possible biases. The providers were assigned to one of the following eight categories: Self-help organization, patient guide, hospitals, audiologists, CI manufacturers/manufacturers of hearing aids, medical publication/physician's guide, other, unclear author.

### Statistical analysis

The results obtained were collected, analyzed using descriptive statistics, and presented graphically in Excel365 (Microsoft, Redmond, Washington, United States) for a better overview. The values were expressed as mean ± standard deviation with minimum (min.) and maximum (max.). The dependence of the complexity of the texts (points in the Flesch Reading Ease Score) on the technical completeness was statistically assessed in the entire sample. The statistical program SPSS29 (IBM SPSS Statistics 29) was used for this purpose. The Gaussian distribution of the samples was determined using QQ plots and the Kolmogorov–Smirnov–Lilliefors test. A statistical correlation was tested using Spearman's rank correlation coefficients and linear regression. P-values less than 0.05 were considered statistically significant.

## Results

### Identified websites and providers

**Fig. 2:**
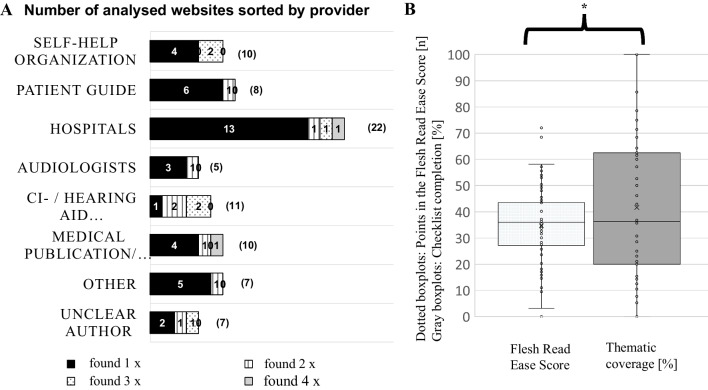
**a** Number of websites found sorted by provider. The number of times the same web pages were found for different search phrases is marked in color. **b** Overall result of all web pages found for the Flesch Reading Ease Score (dotted) and for thematic coverage of the checklists (grey background). *The statistical significance was determined using Spearman's rank correlation (r = − 0.413, p = 0.00014) and linear regression (slope = − 0.93, p < 0.0001)

A total of 80 websites were identified and subjected to further analysis. These 80 websites were created by 54 different providers. A total of 16 websites were represented in the first ten hits for several search phrases (eight were found twice, six were found three times, and two were found four times (Fig. 2a). The allocation to the individual categories of providers (self-help organization, patient guide, hospitals, hearing aid acousticians, CI manufacturers/hearing aid companies, medical publication/doctor's guide, other, unclear author) averaged 8.3 ± 1.98 entries (min. 5; max. 22) per group. The group of hospitals showed 22 hits.

### Thematic coverage

**Fig. 3:**
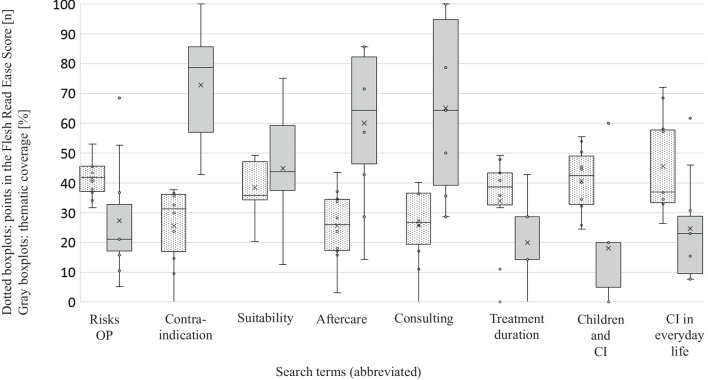
Results of the Flesch Reading Ease Score (dotted line) and the thematic coverage of the checklists (grey background) sorted by search phrase

On average, 41.6 ± 28.2% (min. 0%; max. 100%) of the items for the respective website were found with the checklists (Fig. 2b). There were clear differences between the individual search phrases. Particularly little or incomplete information was found on the topics "duration of treatment" (20 ± 11.4%; min. 0%; max. 42.9%), "CI in children" (18 ± 16.6%; min. 0%; max. 60%), and "CI in everyday life" (24.6 ± 16.8%; min. 7.7%; max. 61.5%). The terms "contraindications" and "suitability for a CI" contained at least 50% of the information (Fig. 3).

**Fig. 4:**
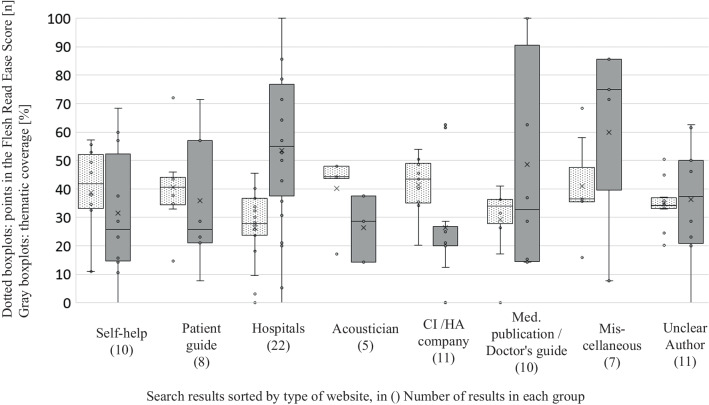
Results of the Flesch Reading Ease Score (dotted line) and the thematic coverage of the checklists (grey background) sorted by provider

Looking at the thematic coverage broken down by the eight provider groups of the websites, the highest levels of completeness were achieved by the groups "Other" (59.9 ± 33.4%; min. 7.7%; max. 85.7%), "Hospitals" (53.6 ± 27.0%; min. 0%; max. 100%), and "Medical publications/doctor's guides" (48.6 ± 36.4%; min. 14.2%; max. 100%). Other provider groups reached between 26 and 36% (Fig. 4).

### Readability with the Flesch Reading Ease Score

On average, the text analyzed achieved a Flesch Reading Ease Score of 34.7 ± 14.2 points (range: 0 to 72) as illustrated in Fig. 2b. Notably, none of the phrases examined attained an average score exceeding 50 points (see Fig. 3). The search phrase "CI in everyday life" exhibited the highest readability, scoring 45.7 ± 15.7 points (range: 26.5 to 72) and was thus the most suitable for readers. In contrast, the phrase "CI aftercare" demonstrated the lowest readability with scores of 25.5 ± 11.6 points (range: 3.15 to 43.7), indicating the lowest suitability for readers (Fig. 3).

Breaking down the data by eight website operator groups, the "Other" group achieved the highest scores with 41 ± 15.9 points (range: 15.8 to 68.4), closely followed by the "CI/hearing aid companies" group with 41.4 ± 9.3 points (range: 20.2 to 53.9) (Fig. 4). In contrast, the "Hospitals" operator group scored the lowest, indicating the least readable content, with 26.4 ± 12.7 points (range: 0 to 45.5) (Fig. 4).

### Statistical analysis

Upon examination, the data set for thematic coverage did not exhibit normal distribution according to both the QQ plot and the Kolmogorov–Smirnov–Lilliefors test. Similarly, the Flesch Reading Ease Score data did not show normal distribution in either test. Spearman's rank correlation (r = 0.413, p = 0.00014) and linear regression (slope = − 0.93, p < 0.0001) were employed to demonstrate a statistically significant negative correlation between the Flesch Reading Ease scores and thematic coverage.

## Discussion

The treatment of a patient with a cochlear implant (CI) requires intense preoperative counseling. This study aims to assess the thematic coverage and readability of information available publicly on the internet regarding CI care. Additionally, we identified interest groups providing this information to the internet.

Firstly, we looked at the thematic coverage of the online information. Substantial variations were observed when analyzing the thematic coverage. Notably, topics such as "contraindications", "aftercare", and "counseling" each achieved a thematic coverage rating of at least 2/3. However, in areas like "CI in children", "duration of treatment", and "risks of surgery," the checklist fulfillment rate was well below 1/3. The shortage of information on CI treatment in children is particularly evident when comparing to other areas. Illustrative of this incomplete information is the fact that only three out of ten analyzed webpages highlighted the necessity of bilateral CI treatment when an indication is given. Moreover, none of the websites, concerning "CI and children", advised that, in addition to audiological criteria, the evaluation of speech development, communicative skills, or the general level of development should also play a role in the decision to treat children. However, as per the CPG and white paper, these two aspects, namely "bilateral fitting" and "assessment of other criteria", are crucial when counseling parents considering CI fitting for their children [2, 13].

The analysis reveals a high diversity regarding the thematic coverage of information found on the internet. Critical areas with more comprehensive information needed include "CI in children", "duration of treatment", and "risks of surgery."

Examining the readability of the analyzed texts, they do not seem to meet the needs of patients. The average Flesch Reading Ease Score falls within the "Hard to read" classification (34.7 ± 14.2 points [min. 0; max. 72]). Overall, only 10% (8 out of 80) of the websites obtained a score of more than 50, being equivalent to "fairly hard to read" or "plain English". No text was classified "Easy to read". These findings align with Sanad et al., who similarly found an average difficulty level of "Difficult" in 103 analyzed websites using the search phrases "cochlear implant" and "cochlear implant in children” [16].

If readability and thematic coverage of the identified websites are related, a significant negative correlation (Spearman's rank correlation coefficient) is evident between lower readability and higher thematic coverage. In simpler terms, texts that have higher thematic coverage tend to be linguistically more challenging. This elevated linguistic level, especially in the context of complex information, poses a challenge for patients. Patients, who may already face limitations in reading or hearing impairments, may judge difficult texts even more challenging. Additionally, the complexity of information could prevent patients from seeking assistance, potentially delaying adequate hearing care.

To address these issues and enhance the readability and comprehensibility of texts for patients, the US Department of Health and Human Services recommends that the difficulty level of medical texts should not exceed 6th-grade level of the US education system [17, 18]. This corresponds to 70–79 points in the Flesch Reading Ease Score or the "plain English" classification. Currently, this recommendation is not met by most of the analyzed websites. It would be beneficial for providers of health-related websites to conduct readability tests, such as the Flesch Reading Ease Score, to better align content with the respective target group (patients).

Until readability is considerably improved, all involved in the CI counseling and treatment process should be aware that patients or their families may face serious difficulties to obtain and understand web-based information. This finding also emphasizes the importance of a personal consultation for CI.

The quality of the internet-based information should be assessed not only on the basis of a subject-related evaluation but also with regard to the providers of the websites. Grouping these according to stakeholder affiliation, each stakeholder group is represented on average by eight websites among the 80 identified websites, with one exception. Only hospitals where CI surgery is performed are represented with an above-average number of 22 search results. What is particularly noteworthy is the difficulty of readability from hospital websites, with an average Flesch Read Ease Score of just 26 points. It is also not clear whether the primary aim of these websites is to provide general information to patients or to advertise the specific hospital to patients. These findings are particularly surprising given that the information provided by hospitals accounts for a quarter of the top ten search engine results on CI. It would therefore be desirable for the providers of these pages to consider results from our study in order to further improve their content.

The provider group "Other" achieved the highest level of thematic coverage with an average of 60%. This high thematic coverage (> 85%) is well explained by two doctoral theses on the topic of CI and one Wikipedia article on the topic of CI, which are allocated to this group.

The provider group of "CI and hearing aid companies" and "acousticians" achieved the best readability in the Flesch Reading Ease Score with 41 and 40 points respectively. Even though these are the best values compared to the other groups, they still correspond to the "hard to read" classification. In the case of commercial providers (CI companies), it can be assumed that the web design is supported professionally and therefore a considerable amount of effort was put into patient information and ultimately customer acquisition. However, the thematic coverage of the information found on these sites is in the lower third compared to the other groups. It can therefore be concluded that, unfortunately, none of the analyzed provider groups met the requirements of a full thematic coverage combined with high readability. This is in line with other studies from the healthcare sector, for example by Bruce et al., who analyzed the quality of online information to support patient decision-making in breast cancer surgery [19].

In this context, however, it can be critically discussed which and to what extent information is necessary or relevant when looking for information. As an example, complications can be mentioned, where there is a potentially long list of items. This conflict of full coverage on information, “practical importance” and readability is a continuous challenge.

Different user groups will have different needs for information content. Thus, one solution may be to differentiate between various levels of detail and information depth for users. This could be adapted to the respective target group, e.g. "general information", "patients", "doctors", "audiological specialists". For example, all CI companies already have different areas for healthcare professionals and patients on their homepages. This approach could also be considered for the websites created by other providers.

It seems indisputable that personal counseling continues to be very important for patients [20, 21]. This is also emphasized by the recommendation of the CI CPG and the white paper [2, 13]. However, consultations could be supplemented by internet-based information, if the content meets the required quality aspects.

### Limitations

One limitation of our study is the used algorithm of the search engine that is based on numerous unknown influencing factors. In order to ensure the most independent internet search possible, all cookies were deleted after each search query and searches were carried out from various PCs, including private PCs that were not connected to the hospital network. However, the search algorithm remains one of Google's central company secrets. It therefore remains unclear at present whether an internet search in another geographical area or at a different time of the day, for example, would have created different results. The results obtained in this study nevertheless appear plausible, as there was a balanced proportion of different providers.

A further limitation is the definition of the search phrases used, as these were defined by the team of authors but not by the patients concerned. In order to counter this limitation, patients could first be asked about their search preferences. At present, however, it is questionable whether potentially different search queries would have yielded in different results. To address this topic a future study may be worthwhile to be conducted.

## Conclusion

In this study, we demonstrated that internet-based information regarding cochlear implant care is highly heterogeneous and often incomplete, as well as difficult to understand for patients. Full thematic coverage of content and readability are mostly unsatisfactory. Relating content towards existing guidelines, simplifying the language using established tools (Flesch Reading Score) and differentiating the information according to the specific needs of the respective target group could substantially help patients during their decision-making process for CI treatment.

## Supplementary Information

**Additional file 1:** Checklists for completeness of thematic coverage.
