# Supplementary material for: Thematic coverage and readability of online patient information on cochlear implant care
Source: Eur Arch Otorhinolaryngol. 2024 May 6;281(9):4727–34. doi: 10.1007/s00405-024-08694-x (PMC11392990; doi:10.1007/s00405-024-08694-x)
Supplement: Supplementary file 1 — Additional file 1: Checklists for completeness of thematic coverage. [file 405_2024_8694_MOESM1_ESM.docx]

**Checklists for completeness of thematic coverage**

Checklist 1: *Who is suitable for treatment with a Cochlear Implant?*

(8 Items)

| Patients who are likely to achieve better hearing and speech comprehension with CI than with hearing aids, bone conduction hearing aids or implantable hearing systems. |
| --- |
| Based on the preliminary examinations, the auditory nerve and auditory pathway can be assumed to be functioning properly. |
| Based on the current state of knowledge, a CI is indicated from a single-syllable discrimination with optimal HG treatment of ≤ 60 % (at 65 dB). |
| For postlingually (after speech acquisition) deafened and residual hearing patients, indication after a long period of deafness can generally be assumed. |
| In prelingually (before language acquisition) deaf (deafened) adults, there is also an indication for implantation in selected individual cases |
| Prelingually deaf and perilingually deaf or residual deaf children |
| If a progressive cochlear-obliterative process (e.g. bacterial labyrinthitis) is suspected, a CI treatment should be performed as early as possible |
| Unilateral hearing loss (SSD) with CI indication with and without tinnitus |

Checklist 2: *Counselling process prior Cochlear Implantation?*

(14 Items)

| The CI-providing institutions should provide personal counselling |
| --- |
| The structure of the CI care facility must have a comprehensive and permanent staffing level to ensure the necessary expertise for patient counselling |
|  |
| *Counselling and information should be provided on:* |
|  |
| Explanation of the care process (pre- and post-operative phase including rehabilitation), |
| Function and mode of action of a CI, various CI systems: The counselling is manufacturer-neutral. It provides the patient with criteria with which they can make a choice for their individual situation, |
| Presentation of alternative forms of care and treatment, |
| Differences to hearing aids and integrability with existing hearing aids, |
| Surgical procedure and risks of the operation, |
| Prospects of success of CI treatment, |
| Clarification and, if necessary, adjustment of expectations, |
| Individual therapy planning, |
| Organising contacts to self-help groups for CI users or parents of CI users, |
| Restrictions and risks of CI fitting |
| Information and contact with those affected, |
| Social law counselling |

Checklist 3: *Treatment duration of a Cochlear Implantation*

(7 Items)

| Implantation: The length of hospital stay is determined by professional medical and social patient criteria. |
| --- |
| Basic therapy begins between the first postoperative day and 6 weeks postoperatively |
| Basic therapy for adults usually takes place under inpatient conditions (3 - 5 days) |
| This is usually followed by 40 days of follow-up therapy (CI rehabilitation) over a period of 6 - 24 months |
| Children: Follow-up therapy (CI rehabilitation) can last until the age of 18 and usually comprises a period of 60 treatment days. Rehabilitation should continue at least until language acquisition and written language acquisition are complete |
| Counselling, surgery, initial adaptation, follow-up therapy and rehabilitation should generally take place within 24 months for adults |
| Lifelong aftercare |

Checklist 4: *Contraindications for a Cochlear Implant treatment*

(7 Items)

| *Absolute contraindications* |
| --- |
| Evidence of a missing cochlea or a missing auditory nerve. |
| The patient is unable to participate in the overall process of CI fitting (e.g. basic therapy, rehabilitation, aftercare). |
| No possibility or no access to initial fitting, rehabilitation or aftercare (patient- or institution-related). |
| *Relative contraindications* |
| Middle ear infections (implantation possible after treatment). |
| Limited ability to undergo rehabilitation after CI treatment. |
| Negative subjective promontory test depending on the results of further audiological diagnostics. |
| Serious concomitant diseases that significantly impair the treatment process |

Checklist 5: *Surgical risks of Cochlear Implantation*

(19 Items)

| Infections of the middle ear |
| --- |
| Wound healing disorder |
| Dizziness and balance disorders |
| Facial nerve paralysis |
| Taste disturbance |
| Tinnitus |
| Loss of any residual hearing |
| Technical implant defect |
| Medical complications due to the implant |
| Need to replace the implant |
| Deterioration of electrical stimulation, e.g. as a result of progressive ossification of the cochlea after meningitis |
| Electrode malposition |
| Undesirable stimulation effects of other cranial nerves (e.g. facial nerve, vestibular nerve) |
| Intolerance to implant materials |
| Haemorrhage; dura and brain injury, cerebrospinal fluid fistula |
| Failure or lack of expected hearing success |
| Neuralgia, scar pain |
| Anaesthesiological risks |

Checklist 6: *Aftercare of in Cochlear Implantation*

(7 Items)

| An existing CI treatment requires lifelong follow-up care (long-term follow-up care) under the responsibility of the institution providing the CI. |
| --- |
| Aftercare is used for medical, audiological, hearing and speech therapy as well as technical monitoring and counselling, including documentation (long-term effects) |
| The aim of aftercare is to stabilise and optimise the individual's ability to communicate. |
| Follow-up care serves to ensure the results of therapy and quality assurance, as well as to determine the indications for additional diagnostic and therapeutic (possibly rehabilitative) measures. |
| Annual check-up |
| Aftercare can be carried out on an inpatient, day-care or outpatient basis. It should be carried out at least once a year for adults. |
| Follow-up care for children generally requires closer examination intervals (usually twice a year) and a longer time frame |

Checklist 7: *Cochlear implantation in children*

(5 Items)

| For postlingually deaf children and adolescents the audiological CI indication should be fulfilled if the monaural single-syllable comprehension measured with a hearing aid in the free sound field at a speech level of 65 dB SPL is ≤ 60% |
| --- |
| For children with a hearing loss > 70 dB HL, the prerequisite for CI fitting should be assessed, taking into account the audiological criteria as well as the assessment of speech development, communicative skills, the general level of development and socio-familial aspects |
| If a bilateral CI is indicated, bilateral implantation (simultaneous or sequential) should be carried out |
| In infants with confirmed bilateral deafness, CI fitting should be carried out in the first year of life if indicated ( |
| In adults and children with SSD (single-sided deafness) or AHL (asymmetrical hearing loss with unilateral deafness and hearing loss on the opposite side), a cochlear implant should be recommended. |

Checklist 8: *Cochlear Implants in everyday use*

(13 Items)

| X-ray at the dentist possible |
| --- |
| CIs can lead to shadows on the head during MRI: possibly choose an alternative diagnostic option |
| MRI is possible (1.5- 3 Tesla) |
| External parts: protect with a helmet during sports such as skiing/ inline skating, protect from the cold with a cap/headband in winter (batteries also last shorter in the cold) |
| Swimming/swapping: remove the sound processor or protect it with a cover, |
| Internal implant: Diving up to 40 metres is possible. |
| Cinema/theatre/lectures: partly inductive hearing systems - interference-free signal transmission via telecoil |
| Telephoning: modern CIs can be connected directly to a smartphone for telephoning/listening to music |
| Wireless accessories are available for older CIs |
| Getting used to/training until telephoning works well takes time |
| Travelling: remove the sound processor at airport security checks |
| Switch processors designed for wireless data transmission to flight mode |
| Spare parts, such as a replacement sound processor, belong in every suitcase, as well as a copy of the MAP issued by the audiologist and a list of clinics in the area in case of emergency |
